# False Impressions

**Published:** 1888-12-15

**Authors:** Caroline Fothergill

**Affiliations:** Author of "An Enthusiast," "A Voice in the Wilderness," etc.


### False Impressions.

By Caroline Fothergill, Author of "An Enthusiast," "A Voice in the Wilderness," etc.

( *Continued from p.* 158. )

### Chapter XI.—Discontented.

As soon as Beatrice's winter engagements were over, she and Mrs. Ledward left Mayfield and went on to the Continent for a time; then, as the spring advanced, they came back to England and settled in the country for a month or two. Beatrice felt that change of scene was absolutely necessary to her. She was more discontented and "out of conceit" with herself than she had ever been before, and she had altogether treated herself with a rigour which poor little Edith under similar circumstances would have been as unable as she would have been unwilling to apply to herself. She was thoroughly tired of Mayfield, and but for her social work, which was too interesting and satisfactory to be willingly given up, she would have left the town for good and all when she went to Paris. Even her social work had failed to satisfy her lately; in fact, she had been thoroughly at war with herself, and the feeling had shown itself in an attack of irritability, happily as brief as it had been severe. She felt better as soon as she got out of town, and on Laura's remarking the change, had said with an expression which betokened that she was ashamed of herself, that she thought her liver must be upset. Mrs. Ledward had made a becoming reply, mentally wondering what could possess Miss Stanford to lay claim to so undesirable a possession as a liver and its necessary penalties; a possession, moreover, which she had hitherto always repudiated with scorn.

They enjoyed France, and stayed there some time, and when they got to the English lakes in an exceptionally balmy May, Beatrice seemed to revive altogether. They settled at Coniston, at Sly's Waterhead Hotel, and Beatrice was never weary of wandering about the exquisite country. She spent all her time out of doors, taking long mountain walks, often alone, for Laura was no great pedestrian; sitting in the sunshine gathering flowers, and sending boxes of them to her Mayfield friends. She spent hours in thought, too—silent, apparently concentrated thought—and Laura noticed that after one of these "attacks," as she called them, Beatrice had always a fit of restlessness and energy, which took a little time to work off. Laura herself was very much interested in her friend just now, although Beatrice did not take her into her confidence. She felt sure that she knew what it was all about, and got quite excited from time to time at her own thoughts. She never dared mention what was in her own mind to Beatrice, but she went as near to it as she dared.

"How long are you going to stay away, Beatrice?" she asked one day.

"I don't know," was the reply; "perhaps until the end of June."

"That seems an odd time to leave the country. It will be beautiful here then, and Mayfield will be odious."

"We can go away again, later. I want to be in Mayfield in July."

"Why?" asked Laura, boldly.

"I want to see some of the people I know again—the working people, I mean."

Laura pulled a face. She was not fond of the working people, and had never sympathised with Beatrice's intercourse with them.

"Are you going to work again next winter as you did last?" she asked, after a pause.

"I don't know; I have not made up my mind yet."

"I hope you won't. You do too much; it is not good for you."

"I probably shall not work so much, but it will not be because I am afraid of my health."

"Oh, no; of course not," said Mrs. Ledward comically, "I know it is sacrilege to cast a doubt upon your physical powers, but you are only human flesh and blood when all is said and done."

Beatrice said nothing, and Laura went on—

"Shall you begin as early? When shall you settle down in Mayfield for the winter?"

Beatrice made a movement of restless impatience.

"Oh, don't talk about the winter yet," she said. "We have only just finished one; it is too soon to be looking forward to another. I have not begun to think of it. I may be dead by then, or the world may have come to an end."

Laura stared. It was not usual for Beatrice to express herself in this way; it was more in Mrs. Ledward's own style; but she said nothing. She had known Beatrice ever since she was a baby, and knew exactly how to "take" her. When she thwarted or irritated her, it was because for some, no doubt excellent, reason she chose to do so, not because she knew no better.

Beatrice kept her word. At the end of June they went home, and did not find Mayfield odious at all. The spring had been very late there, and the summer months were cool. The trees were fresh and green, the gardens gay, the air was not stuffy. Life, on the whole, was very bearable there.

Beatrice was full of occupation at once. She visited her friends and frequently went to the museum. Most of the committee were away, and the attendances at the meetings were small. She went regularly; she was carrying out a compact she had made with herself, and there was also a little bravado in what she was doing. She saw George every time, but so far had succeeded in avoiding any conversation with him. On one occasion, however, her plans were overthrown with far-reaching results.

There had been very little business. The meeting, at which Beatrice had been the only lady, was soon over, and she left the room while the others were still talking. She walked slowly down the corridor, stopping every now and then to look at something which caught her notice. Before reaching the staircase she turned aside into one of the rooms, and after looking at what she was in search of, went forward to the window, and stood looking out.

The room, like almost all the others, was at the back of the house. Only the corridor and one or two of the rooms had windows to the front; the majority commanded the prospect upon which Beatrice was looking now—an open space with heaps of rubbish and old iron upon it, a waste of tall chimneys vomiting smoke, and lower ones sending forth sheets of flame; mills of different kinds, rows of dismal "artisans' dwellings," with or without back yards, completed the prospect. Out of town it was a glorious summer day; even in town the sun shone and the sky was blue; but here, in the heart of the working part of the town, summer was as much misery as enjoyment. The sky was obscured by thick clouds of smoke, till it was grey rather than blue; the sun beat down relentlessly and with a kind of coppery glare upon the hard and dirty pavement, the air was stiflingly thick and close, the smells were intolerable. Everywhere dirt, smoke, and hideousness—mental, moral, and physical, human and material. So at least it seemed to her in her present depressed state of mind. She sighed as she looked at it all, and for a few moments lost all faith in the good of what she was doing down here, a drop in the ocean, a footprint in the sand before the advancing tide. After a while she recovered a little and went out of the room again.

At the head of the staircase, just round the corner, so that she had not seen him as she came along, stood George Murray, examining a new picture which had been hung only the day before. She bit her lips as she caught sight of him, and realised that a meeting was inevitable. Nevertheless, she would have passed him without speaking, but he looked up at the sound of her footstep and said:

"Will you give me your opinion about this picture, Miss Stanford?"

She experienced an odd shock of surprise when he addressed her, but somehow, impelled by some "force not herself" she did stand still, although unwillingly, and replied:

"I know very little about pictures, I think you had better get the opinion of someone else, a more competent judge."

Having said this she would have gone on, but he persisted in what he wanted, and went on:

"I think you can tell me this. I would not trouble you with it, but everyone else has gone, and we have waited so long for this picture, I am unwilling to delay the hanging of it any longer. I only want to know if you think this space on the wall which just holds it, allows it to be seen to advantage. I think myself it looks crowded, and that takes away from the effect; but I have been looking at it so long I can no longer see truly."

"You flatter me in implying that I can," she answered, but she did what he asked her; looked at the picture for a little while, standing at various distances from it, and finally gave her verdict against the present position of it.

George agreed with her, and she went on downstairs, but to her annoyance he came too, and they left the museum side by side.

They walked down the garden together, and turned into the street. Here Beatrice stood still. It was one of her fancies regarding this place that she never brought her carriage down here. She either walked or rode in a tram. This afternoon it was too hot to walk, and she intended to take a tram, but they only passed at intervals of ten minutes. There was not one in sight, and in looking at her watch she found she had just missed one.

"Oh," she said aloud, "I have timed it badly. Do not let me keep you," she concluded, turning to her companion.

"If you will allow me I will wait with you," he said. "I am going to take a tram, too."

She could not prevent his doing this, but she at once began to cast about in her mind for some device by which this long tram drive together might be avoided. While she was so occupied, they walked slowly along the street together talking. It was a street very representative of that part of the town—squalid houses, shabby shops, dirty women gossipping at the doors, clamorous children playing in the gutters. As she saw them the thought again came into Beatrice's mind, "How can they be as good as they are, living in such surroundings?"

As they went slowly along an expedient by which her purpose might be served occurred to Beatrice, and she stood still, saying:

"I shall not wait for the tram. Since I am down here I will go and see a friend of mine, of whom I have had no news for a long time."

"Will not your errand be thrown away? You are scarcely likely to find him at home at this hour," said George, anxious to put obstacles in her way, for he had been looking forward to this very tram ride which she was so wishful to avoid.

"He is out of work," she replied, "or was when I saw him last. At any rate, I can see his wife and hear how they are getting on."

"Where do they live?"

"In Dacre Street."

"That is a very unpleasant street, close by the canal. I hope you will let me come with you."

"On no account," was her somewhat impulsive answer.

"I mean," she went on, as he said nothing, but only looked surprised, "it is not in the least necessary. I have often been alone."

"And have you never met disagreeable people?"

"I have met people who looked very disagreeable, but as they never spoke to me I am unable to say if their manners coincided with their appearance."

There was a tone of raillery in her voice, and as he still said nothing she went on.

"I cannot afford to be timid, even if it were my nature to be so. If I wanted a guard in my walks about here, I should have to stop at home altogether; you would scarcely expect me to go about with a policeman at my heels. Good afternoon," she concluded, holding out her hand.

He did not take it, he disregarded altogether her parting words, and said instead:

"I wish you would let me come with you. It is not a fit place for you to venture in alone."

"I assure you it is not necessary," she repeated a little impatiently; "besides, you do not know these people, I could not take you into their house, especially in the distress in which they are now."

"I do not wish to go in, I would wait outside for you."

Her colour rose at his pertinacity. "You are very considerate," she said, "but I would rather go alone."

He turned, as much as to say, "I will oppose you no longer." She again said "Good afternoon," and then left him.

He stood looking after her with a cloud of anger on his face. She was too self-reliant—too much her own mistress, he thought. There was no womanliness in it. She ought to have accepted his judgment. It was unfeminine to refuse his company in such a place. His mood swung round, and all at once he found himself again judging her as harshly as during his talk about her with Dr. Temple. It escaped his attention that he was now blaming her for possessing the very attributes which on that former occasion he had thought were too lacking.

He watched her until she was nearly out of sight, and then a sudden thought struck him, and he resolved to follow her. At a little distance he would be quite safe, and he felt tolerably certain that she was not the kind of woman to turn her head when she walked. He started off in pursuit on the spur of the moment. He was perfectly safe. The streets were busy at this time, and when she turned into Dacre Street he felt emboldened to somewhat decrease the distance between them. She met a good many very rough-looking men, but he was forced to acknowledge she had been perfectly right as regarded her safety. They all looked at her, but she suffered no annoyance, and presently she stopped at a certain house, knocked at the door, and, after a moment's waiting, was admitted.

*(To be continued.)*

